# Pushing the Envelope in Obstetric Care: A Case Report of Cesarean Delivery in a Parturient with a BMI >100 kg m^−2^

**DOI:** 10.1155/2020/5498584

**Published:** 2020-05-27

**Authors:** Sangeeta Kumaraswami, Itamar Futterman, Suryanarayana Pothula, Geetha P. Rajendran, Ashutosh Kaul

**Affiliations:** ^1^Department of Anesthesiology, New York Medical College, Westchester Medical Center, 100, Woods Road, Valhalla, New York 10595, USA; ^2^Department of Obstetrics and Gynecology, New York Medical College, Westchester Medical Center, 100, Woods Road, Valhalla, New York 10595, USA; ^3^Department of Surgery, New York Medical College, Westchester Medical Center, 100, Woods Road, Valhalla, New York 10595, USA

## Abstract

An increasing number of women with a body mass index (BMI) ≥ 60 kg m^−2^, referred to as super-super obesity, are requiring anesthetic care for labor and delivery. Management of these patients presents obstetric, anesthetic, and logistical challenges. We report our experience in the management of cesarean delivery in a parturient with a BMI of 112 kg m^−2^. Use of epidural anesthesia and performance of a supraumbilical transverse surgical incision with caudal placement of the panniculus resulted in optimal hemodynamic and ventilatory parameters. Effective multidisciplinary planning and communication is key. We present this case to highlight decision-making strategies and elucidate our approach in the management of this complex obstetric case.

## 1. Introduction

Obesity is a global epidemic that is reaching alarming proportions in the pregnant population. An increasing number of patients with a body mass index (BMI) ≥ 60 kg m^−2^, referred to as super-super obesity, are being seen [1], resulting in obstetric, anesthetic, and logistical challenges. We describe management of cesarean delivery (CD) in a parturient with a BMI of 112 kg m^−2^ and emphasize multidisciplinary planning and communication for optimal outcomes. We are unaware of any previous reports describing management of CD in a parturient with a BMI >100 kg m^−2^. Written consent was taken from our patient for publication of this case report.

## 2. Case Description

A 30-year-old parturient G2P1 with height 150 cm and weighing 252.20 Kg was scheduled for an elective CD and sterilization by bilateral salpingectomy at 39 weeks of gestation. Her medical history was significant for gestational hypertension and obstructive sleep apnea with nonadherence to the prescribed noninvasive ventilation. The patient was able to sleep only with head of bed elevation. Although she lived a sedentary lifestyle, she reported good mobility that was currently limited by her pregnancy.

Twelve years ago, she weighed 158 Kg when she underwent a CD for a nonreassuring fetal status, following induction of labor for postdated pregnancy. The surgery was done under neuraxial anesthesia that was accomplished after a few attempts. Two years later, she underwent a laparoscopic gastric banding procedure that resulted in a weight loss of 68 Kg, which she subsequently regained. This was followed by additional weight gain in the following years.

A weight gain of 5.5 Kg was reported during this otherwise uneventful pregnancy. Despite counseling, she declined trial of labor. Multidisciplinary planning involved cardiology, bariatric surgery, and the pulmonology service for potential perioperative noninvasive ventilation. A 2D echocardiogram showed normal left and right ventricular systolic function. Her physical examination was notable for a nonreassuring airway and a large panniculus, with inability to palpate the lower thoracic and lumbar vertebral spinous processes. On the day before surgery, she underwent ultrasound-guided insertion of a double-lumen peripherally inserted central catheter (PICC) to provide reliable intravenous access. A preprocedural spinal ultrasound was performed. Though we were able to delineate the midline, we were unable to identify key ultrasonographic structures that would aid in the measurement of the depth to the epidural space.

On the day of surgery, placement of an 18-gauge peripheral intravenous catheter was easily accomplished. Radial artery cannulation was done preoperatively using ultrasound. Since the surgical plan involved a transverse abdominal incision, we elected to place a low-thoracic epidural catheter for surgical anesthesia in the preoperative area. The patient experienced difficulty in autopositioning herself for the procedure. Additional personnel were needed to facilitate in-bed movement and place the patient in a flexed sitting position for neuraxial placement.

Using the Tuffier's line and scapula as anatomical guides, we placed a multiorifice epidural catheter on the second attempt at the T11-T12 interspace without complications. The epidural space was located at a depth of 12 cm using a 17 G 12.5 cm Weiss epidural needle, and the catheter was threaded 5 cm into the space. A midline approach and a loss of resistance to air technique was used. Following uneventful injection of a 3 ml test dose of 2% lidocaine with 1 : 200,000 epinephrine, we taped the catheter in the relaxed sitting position. About 20 minutes after injection of 100 mcg epidural fentanyl and 900 mg 3% chloroprocaine that was administered in divided doses, the patient reported development of a tingling sensation in her legs. She was then transferred to the operating room.

**Figure 1:**
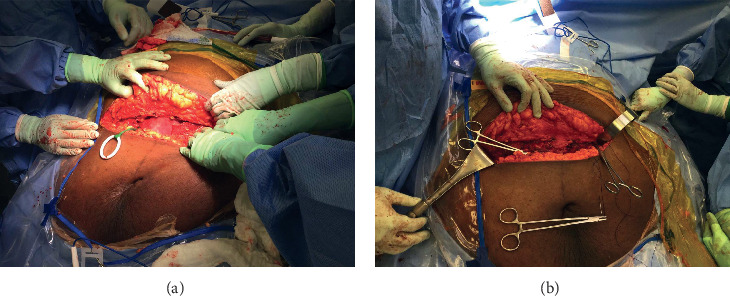
(a) Supraumbilical transverse surgical incision 2-3 cm above the pubic symphysis. (b) Wide surgical incision extending beyond the midclavicular lines bilaterally, with caudally placed panniculus.

After transfer to the bariatric table aided by a HoverMatt air mattress®, we first placed the patient in a ramped position with left uterine displacement. At the surgeon's request, a 15° reverse Trendelenburg position with abduction of her legs was achieved, so that a part of the panniculus could be accommodated between her legs. An area for a transverse incision was marked 2-3 cm above the pubic symphysis. Since the umbilicus was now displaced downwards by the panniculus to the level of the pubic symphysis, the planned incision corresponded to an estimated T8 dermatomal level. Three grams of cefazolin, a dosage recommended for patients ≥120 Kg was administered intravenously for antibiotic prophylaxis [2]. After additional epidural boluses of 3% chloroprocaine, we confirmed a sensory block to the T5 dermatomal level. The surgery commenced without patient discomfort (Figures 1(a) and 1(b)).

Following a transverse skin incision that was extended beyond the midclavicular lines bilaterally, the surgical team proceeded to dissect the layers of adipose tissue in the upper part of the panniculus. Intraperitoneal adhesions were subsequently encountered presumably secondary to the previous cesarean delivery. An hour after skin incision, a neonate weighing 3040 gm with Apgar scores of 8 and 9 at 1 and 5 minutes was delivered through a transverse hysterotomy. A transverse uterine incision was preferred due to potentially less blood loss and easier reapproximation than a vertical incision.

Increased bleeding was encountered after delivery of the neonate. An infusion of 1000 ml 0.9% normal saline containing 40 IU oxytocin was initiated at the obstetrician's request, without further need for additional uterotonic agents. 1000 mg tranexamic acid was given intravenously over 10 minutes [3]. Two units of blood were transfused, and 3 grams of cefazolin was redosed secondary to a blood loss greater than 1500 ml [2]. Transient decreases in blood pressure were supported by phenylephrine boluses. We administered 3% chloroprocaine intermittently every 30 minutes through the epidural catheter with satisfactory surgical anesthesia. Prior to wound closure, the surgical team examined the segment of the patient's gastric band tubing that was accessible via the incision. The tubing was confirmed to be anatomically intact. The patient experienced transient discomfort during this surgical manipulation. 2 mg of midazolam was then administered intravenously with transient oxygen desaturation to the eighties. The surgery was completed uneventfully with an estimated blood loss of 2000 ml. She received supplemental oxygen through a nasal cannula during the 4-hour procedure (operative time 3 hours). A total of 6000 mg 3% chloroprocaine with 300 mcg fentanyl was administered epidurally until the end of surgery. Intraoperatively, our patient remained hemodynamically stable, barring occasional use of short-acting vasopressors (Table 1).

Postoperatively, epidural analgesia was initiated with 0.2% ropivacaine infusion and continued for 18 hours. During this time, she remained in the ramped position and received supplemental oxygen through the nasal cannula. She continued to receive mechanical venous thromboembolism prophylaxis with pneumatic compression devices. Pharmacological venous thromboembolism prophylaxis was initiated with subcutaneous unfractionated heparin 5000 units thrice daily. The following day, her epidural catheter was removed and she began to ambulate. She was discharged home on the third postoperative day after being transitioned to a 6-week thromboprophylaxis regimen of subcutaneous low molecular weight heparin 100 mg daily. Her postpartum course remained uneventful. She was counseled regarding weight management interventions prior to discharge.

## 3. Discussion

Care of parturients with super-super obesity remains challenging, with high rates of maternal complications at delivery [4]. Considerations include a thorough preoperative assessment and optimization, appropriate choice of surgical and anesthetic techniques, perioperative drug dosing adjustments including venous thromboembolism prophylaxis, and availability of additional personnel and specialized bariatric equipment.

Difficulties with intravenous access, blood pressure monitoring, positioning, insertion of neuraxial blocks, and airway management were the expected perioperative challenges [5]. Reliable large-bore intravenous access was necessary; however, traditional central venous cannulation can be associated with difficulties in insertion and risk of complications. We opted for a PICC due to potentially easy placement, decreased risk of dislodgement, and ability for prolonged vasopressor administration [6]. The placement of an 18-G cannula ensured large-bore peripheral venous access. Arterial cannulation was considered essential due to anticipated difficulty with noninvasive blood pressure monitoring and potential for hemorrhage.

Neuraxial techniques are the preferred mode of anesthesia for CD due to an increased risk of complications with general anesthesia [7]. Spinal anesthesia, epidural anesthesia, combined spinal-epidural anesthesia (CSEA), continuous spinal anesthesia, and double neuraxial catheter techniques (epidural catheter with CSEA or epidural catheter with a continuous spinal technique) have all been successfully used as anesthetic techniques in obese parturients [5, 8–10]. A continuous technique is ideal with higher BMI [1, 11], conferring the ability to extend a neuraxial block. Increased likelihood of multiple attempts, accidental dural puncture, and epidural vein cannulation exist with neuraxial placement [7, 8].

Epidural anesthesia offers several advantages including an easily titratable local anesthetic dose and level of anesthesia, ability to extend the block if the surgery gets prolonged, slower and more easily controllable hemodynamic changes, and utilization of the catheter for postoperative analgesia [12]. Disadvantages of spinal anesthesia include the time-limited nature of the block. A dense T4 level of spinal anesthesia may cause intraoperative difficulties with ventilation [13]. Disadvantages of CSEA include risk of an untested catheter and inadequate surgical anesthesia or postoperative analgesia for thoracic dermatomes [5]. A continuous spinal technique may result in a postdural puncture headache [7]. Double neuraxial catheter techniques may be beneficial for high vertical supraumbilical incisions [1]. As BMI increases, optimal image quality with ultrasound may be difficult to obtain [1]. Although we were unable to estimate the depth to the epidural space, identification of the midline by ultrasound did increase our confidence prior to epidural placement.

The epidural catheter was placed before proceeding to the operating room. With increased risk of epidural failure in this patient population [14, 15], we planned early block assessment to allow for backup plans. Good positioning is essential to optimize neuraxial placement. Verbal communication with the patient can be useful in identifying the midline [16]. Neuraxial techniques may be technically easier in the sitting flexed position, rather than the lateral position in this patient population [1]. Adhesive tape may be used to retract the lateral pads of fat from the midline [7].

Following epidural placement, the patient should be allowed to return to a relaxed sitting position before securing the catheter to the skin, important especially in obese patients. When the patient is in the sitting position and flexion of the lumbar spine is optimized, the distance from the skin to the ligamentum flavum is minimized [7]. On returning to a relaxed sitting position this distance increases, and the skin and soft tissues may move caudad. If the catheter were to be secured to the skin before the patient is allowed to return to a relaxed position, the catheter may be pulled back out of the epidural space by the distance that the soft tissues travel when returning to this position, even as the catheter mark at the skin stays constant. This could lead to complete failure of the epidural catheter if not recognized. Consideration may also be given to allowing patients to lie in the lateral position prior to securing the catheter, as this could allow the soft tissues to move even further.

With increased time being required to position the patient and longer neuraxial procedure times [1], performance of the block in the preoperative area may contribute to operating room efficiency [17]. A possible disadvantage is catheter dislodgement during patient movement [15]; however, transfer with an air mattress and adequate catheter length in the epidural space reduces this possibility [18].

The patient expressed concerns regarding her ability to lie recumbent during the procedure. A ramped position aided by the reverse Trendelenburg position permitted patient comfort, favorable ventilation dynamics, and optimal positioning needed to secure the airway [18]. Supplies for noninvasive ventilation and difficult airway equipment were available for respiratory support or inadequate surgical anesthesia [7].

Specialized bariatric equipment should be readily available for the care of this patient population. These include operating tables, beds, and stretchers with appropriate weight limits. Use of operating table extenders should be considered to support redundant tissue and prevent skin or tissue injury [1]. To reduce risk of injury to personnel, additional staff and availability of specialized positioning devices and air-inflated mats is essential during positioning and transfer of these patients. The staff should be appropriately trained in the use of such devices.

Aortocaval compression in the supine position occurs during late pregnancy and may be relieved by a pelvic tilt, important especially after neuraxial anesthesia [7, 19, 20]. This compression may be further increased in obese patients, particularly those with a large panniculus [19]. The combination of the pelvic tilt combined with the reverse Trendelenburg position may contribute to reduction of aortocaval compression in obese pregnant women [21], and likely helped with the maintenance of hemodynamics in our patient.

Key surgical decisions that impact anesthetic management during CD in the super-super obese parturient include type of skin incision and the positioning of the panniculus. A low transverse or Pfannenstiel incision above the pubic symphysis is commonly done for cesarean sections. However, performance of this incision under a large panniculus may be technically challenging with concerns for suboptimal exposure and wound infection [22, 23]. A vertical abdominal incision has been associated with better visualization; however, disadvantages include an increase in operative time and blood loss with concerns for wound infection, as well as postoperative pain and atelectasis due to diaphragmatic splinting. Though both transverse and vertical abdominal incisions above and below the umbilicus have been described [22–28], the optimal surgical incision remains controversial.

A transverse incision was planned in our patient; however, appropriate positioning of the large abdominal pannus presented a challenge. Vertical, cephalad, or caudad retraction of the panniculus to facilitate optimal surgical exposure has been done using tape and specialized retraction devices [5, 7, 8, 23]. Manipulation of the pannus can cause maternal-fetal morbidity and mortality, secondary to hypoxia and hypotension with displacement of the diaphragm and aortocaval compression [23, 28, 29]. Angled suspension of the panniculus has been suggested to minimize this risk [8]. In our patient, gravity was used to position the panniculus. The ramped position combined with the reverse Trendelenburg position resulted in a natural caudad placement of the panniculus. The positioning of the panniculus away from the surgical field resulted in optimal surgical visualization. The avoidance of pannus manipulation averted any possible cardiopulmonary compromise.

Limited studies guide optimal neuraxial dosing in this patient population [7]. Pregnancy has been reported to enhance the sensitivity of nerves to local anesthetics and to decrease anesthetic requirements during regional anesthesia [30]. Obese patients have reduced epidural space volumes due to increased intra-abdominal pressures when compared with normal patients [31]. Smaller amounts of epidural local anesthetic may then be needed to provide sufficient analgesia or anesthesia. Evidence examining longitudinal epidural spread in pregnancy is conflicting because there is no practical or reliable quantitative measure of spread of the solution in the epidural space [32]. The site of injection of the local anesthetic is the most important variable in determining the segmental anesthetic coverage, with spread occurring in both the cephalad and caudad directions. A volume of 1-2 ml of local anesthetic per dermatome is typically used.

We used the local anesthetic 3% chloroprocaine due to its rapid onset of action. The initial dosing through the epidural catheter allowed timely recognition of epidural functionality. 3% chloroprocaine is also associated with a minimal risk of systemic toxicity secondary to an extremely high rate of metabolism in maternal and fetal plasma [33]. With subsequent redosing, the required surgical level was achieved without complications. Simultaneous administration of epidural fentanyl contributed to an increased dermatomal level and local anesthetic sparing [31].

Increased operative time and blood loss were expected [10, 23] secondary to the increased BMI, a risk factor for postpartum hemorrhage [34]. Because the patient had increased bleeding after delivery of the neonate, we initiated blood transfusion intraoperatively. Oxytocin is considered the first-line uterotonic agent in the prevention and treatment of postpartum uterine atony. There is considerable variation in clinical practice with regard to the optimal dose and rate for use in cesarean sections [35]. The common practice is the continuous infusion of oxytocin at doses greater than 20 to 40 IU [35, 36].

Postoperatively, pain relief may be satisfactorily achieved with continuous epidural analgesia, without respiratory complications related to atelectasis or administration of long-acting neuraxial opioids such as preservative-free morphine [8]. A multimodal analgesic regimen can optimize postdelivery analgesia, decrease opioid consumption, and encourage ambulation.

Venous thromboembolism is a leading cause of maternal morbidity and mortality [37]. Mechanical and pharmacologic thromboprophylaxis should be initiated as early as possible in these patients [1], with a goal for early mobilization. Due to absence of risk factors such as prior thromboembolism events, thrombophilia, or a family history of thromboembolism, our patient was not considered a candidate for antithrombotic drug therapy prior to delivery. Pharmacological prophylaxis was continued for 6 weeks postpartum in our patient due to a high risk for thromboembolism, secondary to presence of risk factors such as obesity and history of cesarean delivery [38].

## 4. Conclusion

**Figure 2:**
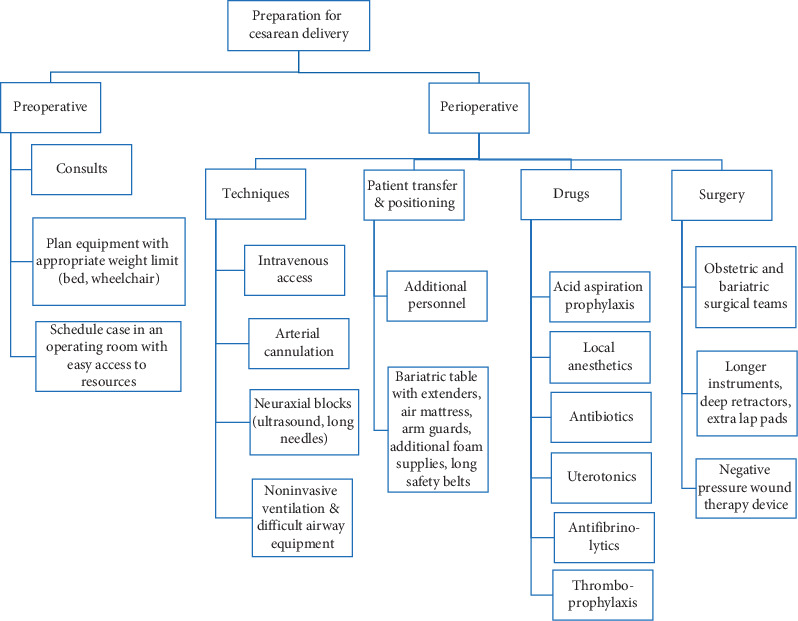
Multidisciplinary planning and preparation for cesarean delivery in our patient.

Obesity is a systemic disease associated with multiple comorbidities [1]. Practitioners must be knowledgeable about the peripartum implications of obesity. Procedures may be technically difficult with risk of complications. Therefore expertise, planning, and preparation is necessary (Figure 2) [14, 15]. Despite the increased risk of morbidity and mortality, our patient had an optimal outcome. An awareness of the respiratory and hemodynamic benefits of the above surgical and anesthetic management will benefit parturients with super-super obesity.

## Figures and Tables

**Table 1:** Perioperative time course of physiological variables.

Time after start of procedure	pH	PCO_2_ (mmHg)	PO_2_ (mmHg)	Hb (gm/dL)	BE (mmol/L)	HCO_3_ (mEq/L)	Lactate (mmol/L)
15 minutes	7.38	38	147	9.7	−2.4	22.5	0.8
After 1 hour	7.38	33	159	8.7	−5	19.5	0.7
After 1.75 hours	7.36	38	178	9.9	−3.6	21.5	0.9
After 6 hours				10.2			
After 24 hours				8.8			

Hb: hemoglobin; BE: base excess; HCO_3_: bicarbonate.
